# Early-Life Respiratory Infections in Infants with Cow’s Milk Allergy: An Expert Opinion on the Available Evidence and Recommendations for Future Research

**DOI:** 10.3390/nu13113795

**Published:** 2021-10-26

**Authors:** Alessandro Fiocchi, Jan Knol, Sibylle Koletzko, Liam O’Mahony, Nikolaos G. Papadopoulos, Seppo Salminen, Hania Szajewska, Anna Nowak-Węgrzyn

**Affiliations:** 1 Translational Research in Pediatric Specialities Area, Division of Allergy, Bambino Gesù Children’s Hospital, IRCCS, Piazza Sant’Onofrio 4, 00165 Rome, Italy; alessandro.fiocchi@allegriallergia.net; 2 Danone Nutricia Research, 3584 CT Utrecht, The Netherlands; Jan.Knol@danone.com; 3 The Laboratory of Microbiology, Wageningen University, 6700 HB Wageningen, The Netherlands; 4 Dr von Hauner Kinderspital, University Hospital, LMU Klinikum, 80337 Munich, Germany; sibylle.koletzko@med.uni-muenchen.de; 5 Department of Pediatrics, Gastroenterology and Nutrition, Collegium Medicum, University of Warmia and Mazury, 10-719 Olsztyn, Poland; 6 Department of Medicine, School of Microbiology, APC Microbiome Ireland National University of Ireland, T12 K8AF Cork, Ireland; liam.omahony@ucc.ie; 7 Division of Infection, Immunity and Respiratory Medicine, University of Manchester, Manchester M13 9WL, UK; ngpallergy@gmail.com; 8 Allergy Department, 2nd Pediatric Clinic, University of Athens, 11527 Athens, Greece; 9 Functional Foods Forum, Faculty of Medicine, University of Turku, 20014 Turku, Finland; sepsal@utu.fi; 10 Department of Paediatrics, Medical University of Warsaw, 02-091 Warsaw, Poland; hszajewska@wum.edu.pl; 11 Department of Pediatrics, NYU Grossman School of Medicine, Hassenfeld Children’s Hospital, New York, NY 10016, USA

**Keywords:** infection, upper respiratory tract infection, infants, cow’s milk allergy, breastfeeding, microbiota, dysbiosis, infant formula, synbiotic

## Abstract

Acute respiratory infections are a common cause of morbidity in infants and young children. This high rate of respiratory infections in early life has a major impact on healthcare resources and antibiotic use, with the associated risk of increasing antibiotic resistance, changes in intestinal microbiota composition and activity and, consequently, on the future health of children. An international group of clinicians and researchers working in infant nutrition and cow’s milk allergy (CMA) met to review the available evidence on the prevalence of infections in healthy infants and in those with allergies, particularly CMA; the factors that influence susceptibility to infection in early life; links between infant feeding, CMA and infection risk; and potential strategies to modulate the gut microbiota and infection outcomes. The increased susceptibility of infants with CMA to infections, and the reported potential benefits with prebiotics, probiotics and synbiotics with regard to improving infection outcomes and reducing antibiotic usage in infants with CMA, makes this a clinically important issue that merits further research.

## 1. The Prevalence of Infections and Antibiotic Use in Healthy Infants and Children

Infections are a leading cause of morbidity and mortality in infants and young children [1], with acute respiratory infections presenting a considerable health burden in developed countries. Upper respiratory tract infections in young children account for more than one-third of paediatric consultations in primary care, according to data for the UK [2] and the USA [3], posing a major burden on healthcare services, as well as stress to parents. The highest incidence rates of respiratory tract infections (RTIs) occur during the first two years of life. A US study showed these young children have an average of six to eight RTIs each year [3]. An Australian birth cohort study in 154 unselected healthy infants found that they experienced an average of 13 discrete acute respiratory infections episodes (incidence rate of 0.56 per child-month) and almost 5 months of respiratory symptoms during the first 2 years of life [4]. A high rate of infections was also seen in a prospective Danish study in infants born to asthmatic mothers, with a median of 14 (range: 2–43) infectious episodes reported during the first three years of life although there was substantial variation in frequency between individuals [5]. In this study, RTIs were the most frequent, with a median of 10 episodes per child, representing nearly three-quarters (71%) of all infections.

Population data from high-income countries suggest that approximately one-quarter of all children are hospitalised at least once with an infection by the age of five years, and one in ten have multiple hospitalisations [1].

Overuse of antibiotics is a global problem, especially in paediatric care. Antibiotic use for acute RTI remains common even if they are largely viral. Antibiotics were prescribed in nearly one-quarter of RTI episodes (21.9%), where a burden diary was submitted by parents in the Australian study [4] and in a similar proportion (24.9%) of infectious episodes in the Danish cohort study [5]. Furthermore, antibiotics are also prescribed in more than 30% of asthma exacerbations in children despite antibiotics not currently being recommended in guidelines and demonstrating minimal clinical benefits [6].

## 2. The Prevalence of Infections in Infants and Children with Cow’s Milk Allergy

Cow’s milk allergy (CMA) is among the most common food allergies in early life, with an estimated prevalence in developed countries ranging from 0.5% to 3% at age 1 year [7]. There are limited data on the prevalence and severity of infections in infants with CMA, although some studies in infants with CMA have reported higher rates of respiratory infections. Infants with a predisposition to atopy have delayed maturation of the Th1 response during childhood putting them at increased risk for infection in the first years of life. Susceptibility to infections has been explored in asthma development [8], but there are relatively limited data on this for food allergy. However, a recent retrospective study found that sensitisation to β-lactoglobulin (cow’s milk protein) was associated with a nearly four-fold increased risk of recurrent respiratory tract infections in children under the age of two years [9]. CMA in infancy has also been shown to be associated with recurrent ear infection (otitis media) [10]. A small cohort study comparing school-age cow’s milk-allergic children with controls showed more than twice the risk of recurrent otitis media, defined as at least 15 episodes by the age of 10 years (27% vs. 12%, *p* = 0.009) and adenoidectomy and/or tympanostomy (48% vs. 28%, *p* = 0.0005), although this occurred only in those developing respiratory atopy [10].

## 3. Factors Influencing Susceptibility to Infection

Children experience numerous infections during childhood with a large and unexplained variation in individual susceptibility [5]. Host factors associated with the individual rather than the external environment are important determinants of susceptibility to infection in otherwise healthy infants and children [5]. An increased risk of infection in children under the age of five years is associated with sociodemographic factors, including lower socioeconomic status, older maternal age, being first born and perinatal factors such as birth by Caesarean section, reduced gestational age and mode of infant feeding [1]. Daycare attendance is another major risk factor for respiratory illnesses in children, and the frequency of colds increases with the number of children in the group [11]. Furthermore, seasonality has been identified as an important risk factor, with respiratory infections being more common in winter [5].

The infant microbiome is important in early immune development and immune-related outcomes. The innate and adaptive immune system plays a crucial role in susceptibility to infections, as well as persistence and clearance of infections [12], and this is strongly influenced by the gut microbiota composition. The development of the intestinal microbiota and the immune system is a complex and dynamic process occurring perinatally and during the first years of life [13]. Immediately after birth, stepwise colonisation takes place, forming the microbiota components that are acquired from the maternal microbiota (vaginal, faecal, human milk, mouth, skin and living environment). Host genotype, gestational age, mode of delivery (vaginal vs. Caesarean section), medical practices (particularly use of antibiotics and proton pump inhibitors), geographic origin and cultural traditions, especially diet, profoundly affect microbiota development [13,14].

## 4. Risk Factors for Increased Susceptibility to Infections in Infants with CMA and the Role of Infections in Allergy Development

There are very limited data as to which factors could explain the increased susceptibility to infections in infants with CMA. Available evidence suggests that infants who develop allergic sensitisation in childhood have different compositions of faecal microbiota in the first years of life compared to nonatopic infants and produce less butyrate [15,16]. In longitudinal studies, gut dysbiosis at 3–6 months precedes the development of symptomatic food allergy [17]. Gut microbiome composition at age 3–6 months was associated with milk allergy resolution by age 8 years (PERMANOVA *p* = 0.047), with enrichment of Clostridia and Firmicutes in the infant gut microbiome of subjects whose milk allergy resolved [18].

Changes in gut microbiota composition have been reported in infants with diagnosed CMA [19], and deficits in healthy microbial exposures may underpin ineffective immune responses to pathogen challenge and contribute to subsequent exaggerated tissue-damaging inflammatory responses that necessitate treatment, medication and hospitalisation.

There are many interactions between antiviral and atopic pathways [20]. Some studies suggest that allergic sensitisation alone is an important risk factor for otitis media with effusion, and interactions between allergy and rhinovirus infections are likely to predispose to otitis media [21]. The possible mechanisms by which atopy might influence the development of otitis media include immunological factors and obstruction of the Eustachian tube [10]. Chronic inflammation of the nasopharynx and obstruction of the Eustachian tube may result in an inability to equilibrate pressure in the middle ear cavity, a build-up of negative pressure and, finally, effusion [10].

The increased risk of otitis media in untreated CMA may be explained by the common origin of mucosa and epithelium in the ear and in the airways and by the interactions between antiviral and inflammatory pathways [21]. Epithelial barrier integrity and permeability in the presence of commensal bacteria and invading pathogens is essential for the maintenance of intestinal homeostasis. If this process is impaired, it can result in inflammation and infection [12]. Around 50% of children with CMA have atopic dermatitis, and epithelial dysfunction may increase the risk for infections in the upper respiratory tract or GI tract.

Interestingly, severe infections in early life have also been associated with an increased risk of allergic diseases in later childhood [22,23]. suggesting the possibility of a vicious cycle of allergic sensitisation and infections and their treatment. Infants that develop viral respiratory infections, especially in the lower airway such as rhinovirus, parainfluenza and influenza, are more likely to develop food-associated allergen sensitisation [23], wheezing illnesses and asthma in later childhood [22,24]. The increased risk of developing food allergy is poorly understood but might be explained by increased skin reactivity to food allergens during upper respiratory tract infections (URTIs) [23]. In particular, the combination of viral infections and allergic inflammation seems to enhance the risk of wheezing [24,25]. A prospective study in infants with an atopy family history showed a higher risk for the development of persistent wheeze in infants with more frequent and intense lower respiratory infection and amounts of aeroallergen-specific IgEs present at the time of the LRI episodes [26].

## 5. The Role of Breastfeeding in Preventing Infections

Infections inevitably occur in all young children, but breastfeeding is one important factor that has been shown to be protective against infections in young children. A large study including 28 systematic reviews and meta-analyses of associations between breastfeeding and outcomes demonstrated that breastfeeding was associated with protection against childhood infections, including respiratory tract infections, acute otitis media and diarrhoea in children under two years of age [27]. Children who were breastfed for longer periods had lower infectious morbidity and mortality into the second year of life and protection against otitis media till 2 years of age and possibly beyond compared to those who were not breastfed or breastfed for shorter periods.

A systematic review and meta-analysis, mainly of observational studies in children under five found a reduced risk of hospitalisation for respiratory infections of 57% in infants that were breastfed vs. not breastfed, and the prevalence or incidence of lower respiratory tract infections was reduced by 32% [28]. In addition, a large Australian study showed that formula-fed-only (never breastfed) infants were more likely than breastfed infants to be hospitalised with infection by the age of five years (any type of infection, adjusted hazard ratio: 1.14, 95% confidence interval: 1.11–1.16) [1].

A review of studies conducted in Europe and the USA found that any form of breastfeeding for the first 6 months of life was protective against acute otitis media in children under two years of age. Interestingly, ‘more versus less’ breastfeeding was associated with a 33% reduction of acute otitis media [29]. This suggests that mixed milk feeding, which occurs in a large proportion of infants, is also likely to confirm protection against infections.

In The Lancet review, authors suggested that important imprinting events might be modulated during breastfeeding [27]. These events could be mediated directly or through effects on the infant microbiome. The ability of the microbiome to regulate host responses in infancy depends on individual bacterial species, which modulate immune regulation and T-cell polarisation, as well as metabolic responses and other processes [27,30]. Human milk contains a complex immune system including living, activated leukocytes and epithelial stem cells [31] and a wide variety of bioactive substances with immune-modulatory, anti-inflammatory and antimicrobial properties that provide protection to infants while their immune system matures [30]. For example, human milk contains a large number of indigestible human milk oligosaccharides (HMOs), which function as prebiotics to support the growth of specific bacteria [32]. More recently, it has been recognised that human milk also contains both viable and nonviable bacteria, postbiotics, that can affect colonisation of the infant gut [33,34]. In addition to changes mediated through the gut microbiota, breastmilk components, such as HMOs might directly influence an infant’s epigenetic programming [27,35]. Moreover, the duration of breastfeeding is important, since many of the components of breastmilk persist throughout lactation.

## 6. Strategies to Influence the Gut Microbiota and Improve Immune Outcomes

Breastfeeding is important in the prevention and treatment of diseases associated with aberrant patterns of microbial colonisation, including infections, highlighting the importance of supporting women to breastfeed their babies. The gut microbiota of formula-fed infants is more diverse compared to breastfed infants and dominated by *Bacteroides*, *bifidobacteria*, *staphylococci*, *Escherichia coli* and *clostridia* [36]. Abnormal colonisation patterns have been associated with long-term effects on immune and metabolic homoeostasis. Considering that breastfeeding is the gold standard for infant nutrition, infant formulas that support the establishment of a microbiota resembling that of breastfed infants might improve immune outcomes in infants that cannot be breastfed.

Supplementation of infant formula with prebiotics, human identical milk oligosaccharides (HiMOs), probiotics or synbiotics (traditionally defined as a combination of a probiotic and a prebiotic) has been used as a nutritional approach to optimise immune responses through modulation of the gut microbiota. This seems to be especially relevant in infants with abnormal colonisation patterns, such as infants born via Caesarean section and infants with CMA. In infants with CMA, an appropriate hypoallergenic formula can be used when breastfeeding is not possible or insufficient to help resolve symptoms and support growth and development. Traditionally, hypoallergenic formulas did not contain factors that stimulate gut microbiota development.

**Figure 1:**
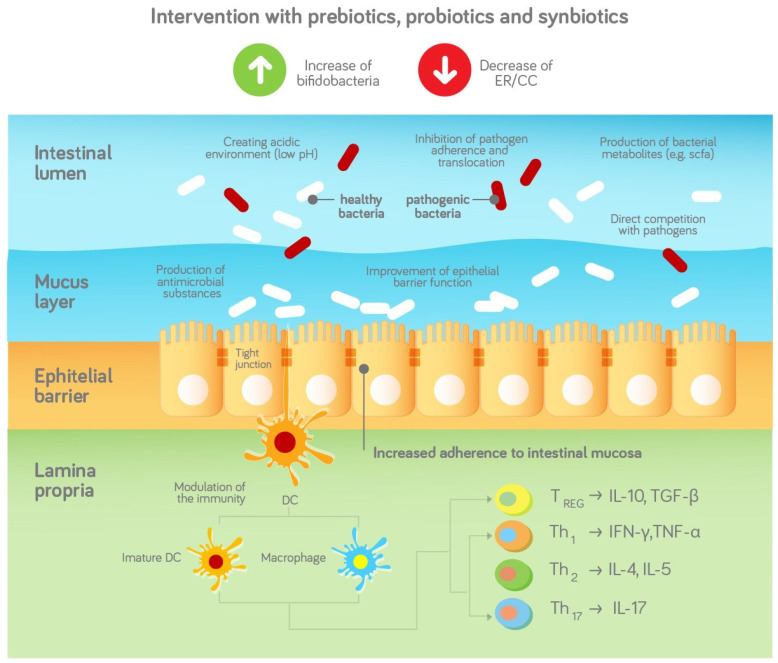
Schematic representation of the effects of prebiotics, probiotics and synbiotics on the gut microbiota and infection outcomes based and adapted from [37], with permission.

Prebiotics and HiMOs are now frequently added to infant milk formula (IMF) to mimic the effects of HMOs and achieve a bifidogenic milieu. Probiotics are live microorganisms that can supplement the bacterial population and reverse the dysbiosis seen in specific target populations. A schematic representation is provided in Figure 1.

Pre-, pro- and synbiotics are added to hypoallergic formula to address the disturbed gut microbiota in infants with CMA and stimulate a bifidogenic milieu. The gut microbiota may help to protect against infections via stimulation of the innate and adaptive immune system. The gut microbiota can also act as a barrier against the infiltration and colonisation of pathogens and therefore protect the infant against infections, e.g., in the following ways [38]

- Competition for adhesion sites and nutrients;
- Production of bacterial metabolites such as SCFA;
- Creating an acidic environment (low pH);
- Production of antimicrobial substances such as antimicrobial peptides;
- Supporting the epithelial and mucosal barrier.

## 7. The Effect of Prebiotics and Synbiotics on Infection Outcomes in Healthy Infants

Several studies looked at the effect of prebiotics, probiotics and synbiotics on infection outcomes. Although the exact mechanism of action is not yet elucidated, it is likely that the immune-modulating effect of prebiotics, probiotics and synbiotics is mediated through intestinal microbiota modification. Oozeer et al. reviewed the effects of IMF supplemented with short-chain galacto-oligosaccharides (scGOS) and long-chain fructo-oligosaccharides (lcFOS) and found that the gut microbiota of infants receiving scGOS/lcFOS was more similar to infants fed with human milk when compared to babies fed with standard formula without scGOS/lcFOS [39].

One of the first studies with scGOS/lcFOS conducted in preterm infants found that infants receiving the IMF containing scGOS/lcFOS had a significantly higher number of Bifidobacteria and a lower sum of clinically relevant pathogens after the intervention [40]. Another study showed that the addition of the specific prebiotic mixture scGOS/lcGOS 9:1 to a partially hydrolysed formula was associated with a reduced risk of upper respiratory infections, a lower cumulative incidence of any recurring infections and recurring respiratory infections [41] and provided a lasting effect beyond the intervention period until 2 years of age [42]. An intervention with IMF containing scGOS/lcFOS in healthy term infants resulted in a lower incidence of gastroenteritis in the supplemented group when compared to the control group [43]. Additionally, fewer infants in the test group experienced more than three upper respiratory infections, and fewer infants had multiple antibiotic courses during the 12 months [43].

A more recent study showed that healthy infants receiving IMF containing GOS and 2′FL had fewer adverse events related to infections and infestations compared to the groups receiving an IMF without GOS and 2′FL or a lower dose of the prebiotics [44]. Infants receiving an IMF containing synbiotics, FOS/GOS and *Lactobacillus paracasei* syp. *Paracasei* showed a significant reduction in the cumulative incidence of lower respiratory tract infections in the synbiotic group compared to the group receiving prebiotics FOS/GOS alone [45]. Fewer cases of parentally reported bronchitis through 4, 6 and 12 months and lower LRTIs through 12 months were also reported in infants that received an intervention with IMF containing 2′Fl and LNnT [46]. These infants also had less antibiotic use through 6 months and less antipyretic use through 4 months [46].

## 8. The Effect of Prebiotics and Synbiotics on Infection Outcomes in Infants with CMA

Fewer studies have been conducted specifically in infants with CMA. Three randomised controlled trials (see Table 1) showed that a specific synbiotic mixture (scFOS/lcFOS and B.breve M-16V) is able to rebalance gut microbiota in infants with CMA and bring it closer in composition and activity to that of healthy breastfed infants [19,47,48].

Several studies have shown fewer respiratory tract infections and otitis media (ear infections) (measured as adverse event outcomes) in infants with CMA receiving a hypoallergenic formula with HiMOs (2′FL and LNnT) [49], as well as with the specific synbiotic mixture scFOS/lcFOS and B. breve M-16V [47]. Synbiotic usage was also associated with reduced overall medication use, including antibacterials and anti-infectives [47,48,50].

## 9. Research Gaps and Recommendations for Future Research

Infections are a leading cause of morbidity and mortality in infants and young children, posing a considerable health burden globally. For future research, it is important to gain a better understanding of the epidemiology of infections in infants and young children, including what might be considered ‘normal’ for the average rate of infections in healthy breastfed infants in order to understand the impact of any intervention, such as nutrition. It is also important to investigate rates of infection in specific groups of children at increased risk in order to explore the impact, underlying mechanisms and potential approaches to reduce the risk of infections. Several studies have shown increased infection susceptibility (especially respiratory infections) in children with CMA, but there is a need for robust, well-designed and conducted studies on the association between CMA and risk of infections. Such studies should use clearly defined criteria for diagnosis of CMA and for infections (differentiating between viral vs. nonviral infection), as well as the indication for any antibiotics prescribed.

Antibiotics are often used inappropriately for viral upper respiratory tract infections. (Over)use of antibiotics has been increasing across the world, contributing to multi-antimicrobial resistance and the emergence of “super bugs”. In infants with CMA, unnecessary antibiotic usage can further disturb the already imbalanced gut microbiota and reduce the potential to fight infections.

In terms of approaches to reduce infections, breastfeeding has been shown to be protective against infections in young children. Supporting breastfeeding is the optimum approach to reducing the risk of infections and associated use of healthcare resources, antibiotic usage and, in severe cases hospitalisations, in infants and young children. It is important to further explore which components of breastmilk can be added to infant formulas that positively influence the gut microbiota and thereby protect against infections. This is particularly important in subgroups of infants at higher risk for infections due to the disturbance of the gut microbiota, including children born after Caesarean section and those treated with proton pump inhibitors or antibiotics. These subgroups may potentially benefit from interventions to support the developing microbiome.

There are currently insufficient data in infants with CMA to determine if there is increased susceptibility to infection, or a more severe response to infectious challenges, but studies have consistently shown a disturbed gut microbiota. Traditional hypoallergenic formulas lack factors that support the gut microbiota and address underlying gut microbiota alterations. Accumulating evidence suggests that supplementing formulas with prebiotics, HiMOs and synbiotics in both healthy infants and in infants with CMA may be associated with reduced risk of infection, as well as lower use of antibiotics, although studies have been limited by infection measures being secondary outcomes. There is currently only one study of HiMOs in CMA, while effects have been shown in three studies in CMA infants receiving synbiotics. Further research is needed to elucidate the mechanism of how the different components in the formula work and protect against infections. The effect of nutritional interventions on infection outcomes in infants with CMA is likely to be a result of microbiome and metabolome modulation, but the mechanism merits further investigation. Studies with synbiotics in infants with CMA show an effect on modulating the gut microbiome in addition to reducing infections, while others with HiMOs in infants with CMA have shown an effect only on the number of infections.

Future studies should investigate both the number and type of infections as a primary outcome. It is essential to clearly define the nature of the intervention being tested, specifying pre-, pro- and synbiotics, and the target population, for example healthy infants or infants with CMA, or other chronic conditions, so that different interventions can be compared across different populations.

Breastfeeding has been shown to reduce the risk of infections in infants and young children, likely due to components in human milk such as bacteria and HMOs. It is therefore important to supplement infant formula with ingredients to bring them as close as possible to human milk. Particularly in high-risk groups, such as those with CMA, it is a clinically important question to further understand the effect of HiMOs, prebiotics, probiotics and synbiotics on reduced infection risk, infection severity and lower antibiotic usage. Well-designed studies are needed to further investigate the mechanisms and clinical effects on infection outcomes in infants with CMA.

## Figures and Tables

**Table 1:** Overview of the effects of prebiotics, HiMOs and synbiotics on the gut microbiota, infection outcomes and medication usage in infants with CMA.

Lead Author (Date)	Intervention	Population (No. of Infants)	Study Outcomes Intervention vs. Control			Description of Effects on the Gut Microbiota, Infection Outcomes and Medication Usage
			Infections	Gut Microbiota	Medication Usage	
Vandenplas (2020) [49] CINNAMON study	Test: EHF with 1.0 g/L2′FL and 0.5 g/L LNnT (HiMO) Control: EHF without 2′FL and LNnT	Infants aged 0–6 months with IgE mediated CMA (194) Diagnosed with CH or 1 symptom + SPT/sIgE/FC	✓	-	-	The odds of infants having at least one LRTI from enrolment to 12 months of age was reduced by 39% in those randomised to formula supplemented with 2′FL and LNnT compared to those given control formula (13/94 vs. 20/96; OR: 0.61 [95% CI: 0.26, 1.40]; *p* = 0.25 N.S.). The odds of infants having at least one URTI from enrolment to 12 months of age was reduced by 9% in those randomised to formula supplemented with 2′FL and LNnT compared to those given control formula (39/94 vs. 42/96/64; OR: 0.91 [95% CI: 0.49, 1.69]; *p* = 0.77 N.S.). Fewer URTI, 60 episodes in 39 infants receiving formula supplemented with 2′FL and LNnT vs. 94 episodes in 42 infants receiving the control formula (corresponding with 0.09 vs. 0.15 episodes/month, HR: 0.58 [95% CI: 0.41, 0.83]; *p* = 0.003).
Burks (2015) [47]	Test: AAF with synbiotics (oligofructose, long-chain inulin*/pAOS, B.breve M-16V) Control: AAF without synbiotics	Infants with IgE or non-IgE mediated CMA aged 0–8 months (110) Diagnosed with DB-FC/CH with sIgE/SPT	✓	✓	✓	Infants receiving formula supplemented with synbiotics had a significantly higher proportion of bifidobacteria (*p* < 0.001) compared with control. The proportion of both C. histolyticum (*p* = 0.009 and *p* < 0.001) and ER/CC (*p* = 0.006 and *p* < 0.001) were significantly lower in group of infants receiving formula supplemented with synbiotics. Fewer overall infections (1 subject (2%) and 10 subjects (18%), *p* = 0.008). Lower use of antibacterials for systemic use (test 17%, control 34%; *p* = 0.049).
Candy (2018) [19] and Fox (2019) [48] ASSIGN study	Test: AAF with synbiotics (oligofructose, inulin/B.breve M-16V) Control: AAF without synbiotics	Infants with non-IgE mediated CMA (122) Diagnosed with CH of ≥1 symptom	✓	✓	✓	Infants receiving formula supplemented with synbiotics had a significantly higher proportion of bifidobacteria (*p* < 0.001) compared with control. The proportion of both C. histolyticum (*p* = 0.009 and *p* < 0.001) and ER/CC (*p* = 0.006 and *p* < 0.001) were significantly lower in group of infants receiving formula supplemented with synbiotics. Fewer overall infections (1 subject (2%) and 10 subjects (18%), *p* = 0.008). Lower use of antibacterials for systemic use (test 17%, control 34%; *p* = 0.049). Lower use of amoxicillin (test 9%, control 32%; *p* = 0.004).
Chatchatee (2021) [50] PRESTO study	Test: AAF with synbiotics (oligofructose, inulin/B.breve M-16V) Control: AAF without synbiotics	Infants with IgE mediated CMA (169) Diagnosed FC/CH with anaphylaxis	✓	✓	-	In the AAF with synbiotics group, the mean percentages of bifidobacteria were significantly higher at 6 and 12 months compared with those in the AAF group (37.1% vs. 6.5%; *p* = 0.001 and 23.9% vs. 6.5%; *p* = 0.026). The mean percentages of ER/CC were significantly lower in the AAF with synbiotics group than the AAF group at 6 months (14.6% vs. 32.6%; *p* = 0.007) but not at 12 months (21.2% vs. 35.7%; *p* = 0.058). Fewer infections requiring hospitalisation (9% vs. 20%; *p* = 0.036) in the AAF with synbiotics group.

EHF: extensively hydrolysed formula; 2′FL: 2′-fucosyllactose; LNnT: lacto-N-neotetraose; pAOS: pectin-derived acidic oligosaccharide; AAF: amino-acid-based formula; *B.breve*: *Bifidobacterium breve*; LRTI: lower respiratory tract infections; URTI: upper respiratory tract infections; ER/CC: *E. rectale/C. coccoides* FC: food challenge; CH: clinical history; SPT: Skin prick test; sIgE: CM-specific serum IgE * Oligofructose, long-chain inulin, also referred to as scFOS/lcFOS.

## Data Availability

Not applicable.
